# Diagnostic performance of semi-quantitative and quantitative stress CMR perfusion analysis: a meta-analysis

**DOI:** 10.1186/s12968-017-0393-z

**Published:** 2017-11-27

**Authors:** R. van Dijk, M. van Assen, R. Vliegenthart, G. H. de Bock, P. van der Harst, M. Oudkerk

**Affiliations:** 1 Center for Medical Imaging, University Medical Center Groningen, University of Groningen, Hanzeplein 1 EB 45, Groningen, The Netherlands; 2 Department of Radiology, University Medical Center Groningen, University of Groningen, Groningen, The Netherlands; 3 Department of Cardiology, University Medical Center Groningen, University of Groningen, Groningen, The Netherlands; 4 Department of Epidemiology, University Medical Center Groningen, University of Groningen, Groningen, The Netherlands

**Keywords:** Magnetic resonance imaging, Coronary artery disease, Myocardial perfusion imaging

## Abstract

**Background:**

Stress cardiovascular magnetic resonance (CMR) perfusion imaging is a promising modality for the evaluation of coronary artery disease (CAD) due to high spatial resolution and absence of radiation. Semi-quantitative and quantitative analysis of CMR perfusion are based on signal-intensity curves produced during the first-pass of gadolinium contrast. Multiple semi-quantitative and quantitative parameters have been introduced. Diagnostic performance of these parameters varies extensively among studies and standardized protocols are lacking. This study aims to determine the diagnostic accuracy of semi- quantitative and quantitative CMR perfusion parameters, compared to multiple reference standards.

**Method:**

Pubmed, WebOfScience, and Embase were systematically searched using predefined criteria (3272 articles). A check for duplicates was performed (1967 articles). Eligibility and relevance of the articles was determined by two reviewers using pre-defined criteria. The primary data extraction was performed independently by two researchers with the use of a predefined template. Differences in extracted data were resolved by discussion between the two researchers. The quality of the included studies was assessed using the ‘Quality Assessment of Diagnostic Accuracy Studies Tool’ (QUADAS-2). True positives, false positives, true negatives, and false negatives were subtracted/calculated from the articles. The principal summary measures used to assess diagnostic accuracy were sensitivity, specificity, andarea under the receiver operating curve (AUC). Data was pooled according to analysis territory, reference standard and perfusion parameter.

**Results:**

Twenty-two articles were eligible based on the predefined study eligibility criteria. The pooled diagnostic accuracy for segment-, territory- and patient-based analyses showed good diagnostic performance with sensitivity of 0.88, 0.82, and 0.83, specificity of 0.72, 0.83, and 0.76 and AUC of 0.90, 0.84, and 0.87, respectively. In per territory analysis our results show similar diagnostic accuracy comparing anatomical (AUC 0.86(0.83–0.89)) and functional reference standards (AUC 0.88(0.84–0.90)). Only the per territory analysis sensitivity did not show significant heterogeneity. None of the groups showed signs of publication bias.

**Conclusions:**

The clinical value of semi-quantitative and quantitative CMR perfusion analysis remains uncertain due to extensive inter-study heterogeneity and large differences in CMR perfusion acquisition protocols, reference standards, and methods of assessment of myocardial perfusion parameters. For wide spread implementation, standardization of CMR perfusion techniques is essential.

**Trial registration:**

CRD42016040176.

## Background

In recent years it has become apparent that information on the functional consequence of a stenosis in the coronary arteries is essential in prognostication and treatment of patients with coronary artery disease (CAD) [1–3]. Invasive coronary angiography is the current gold standard for the assessment of CAD according to the ESC guidelines [4, 5]. Fractional flow reserve (FFR) measurements are used to assess the functional significance by determining the pressure drop over an epicardial stenosis [6]. The disadvantage of invasive coronary angiography is that it is an invasive procedure, exposing patients to procedural risks and radiation [7–11]. In addition, in up to 60% of the patients undergoing invasive angiography, no significant stenosis is present suggesting that the pre-selection of patients for invasive coronary angiography can be improved [12].

A variety of noninvasive imaging modalities exists which show potential to be used in the (functional) assessment of patients suspected of CAD. These modalities include positron emission tomography (PET), cardiovascular magnetic resonance (CMR), computed tomography (CT), and single-photon emission computed tomography (SPECT). The different myocardial perfusion imaging (MPI) modalities all show a high diagnostic accuracy with an area under the curve (AUC) of 0.95 (0.91–0.99) for CMR perfusion imaging in general compared to 0.93 for PET, 0.93 for CT, and 0.82 for SPECT, respectively [13]. A disadvantage of MPI performed with either PET, SPECT or CT is the radiation exposure during the examination [11, 14].

MPI by stress CMR perfusion combines a high spatial resolution with the absence of radiation. These features make CMR perfusion an interesting modality for routine clinical assessment of CAD. The diagnostic accuracy of CMR perfusion imaging has been assessed in multiple studies and recent meta-analyses have provided extensive overviews of available evidence [13, 15–18], however these meta-analyses do not discriminate between qualitative and quantitative assessment. Currently, the visual assessment of perfusion defects is used in clinical practice [19].Visual assessment however, is subjective and highly dependent on expertise. However, analysis of the signal-intensity curves (SI-curves) that can be acquired during the first wash-in of the paramagnetic contrast agent gadolinium have potential to provide quantitative information on myocardial perfusion. These SI-curves to evaluate the myocardial blood flow (MBF) can be evaluated by semi-quantitative or quantitative methods [20]. The semi-quantitative method is based on the maximal upslope of the tissue attenuation curve (TAC) [21]. The quantitative method is based on model dependent deconvolution using the SI-curves. A variety of tracer kinetic models are used providing a MBF value related to the physiological MBF [22]. There are various proposed models for model dependent deconvolution with varying complexity. Both the semi-quantitative and quantitative parameters can be analyzed relatively as a ratio between values during stress and rest MPI or as absolute values. Although a large number of studies have been performed, meta-analysis of CMR perfusion available to date did not evaluate the diagnostic performance of these semi-quantitative and quantitative analysis of the SI-curves acquired during the first-pass perfusion.

Therefore, the aim of this meta-analysis was to assess the diagnostic accuracy of semi-quantitative or quantitative CMR perfusion imaging analysis based on SI time (SI-curves) as compared to either anatomical(quantitative coronary angiography (QCA)) or functional reference standards (invasive coronary angiography +/− FFR) in patients with suspected or known CAD.

## Methods

### Protocol and registration

This meta-analysis was performed in concordance with the Preferred Reporting Items for Systematic Reviews and Meta-analyses (PRISMA) statement and was registered at PROSPERO (http://www.crd.york.ac.uk/PROSPERO/display_record.asp?ID=CRD42016040176) under registration number: 42016040176).

### Eligibility criteria

To produce an extensive overview of the diagnostic accuracy of both semi-quantitative and quantitative CMR perfusion analysis, the following criteria to determine eligibility where used: study domain – patients with known or suspected CAD. Index test – quantitative or semi-quantitative CMR perfusion. Reference standard – invasive coronary angiography +/− FFR and QCA. Study results – diagnostic accuracy of index test compared to reference standard. Study design – observational. Overlap in study population between studies was corrected for by only including the study with the highest number of patients. Studies evaluating visual CMR perfusion outcome measures not based on time intensity curves, evaluation on a segmental basis, animal studies, phantom studies, and dose ranging studies were excluded from both the qualitative and quantitative analysis. Furthermore, reviews and overview documents were excluded from the quantitative analysis.

### Search strategy

The following search strategy was used in Pubmed: (“Myocardial Ischemia”[Mesh] OR myocardial OR cardiac OR “coronary artery”) AND (“Magnetic Resonance Imaging”[Mesh] OR Magnetic Resonance[tiab] OR mri[tiab] OR MRP[tiab]) AND (“Perfusion Imaging”[Mesh] OR perfusion[tiab]) AND (Quantification*[tiab] OR quantitative[tiab] OR deconvolut*[tiab] OR myocardial perfusion reserve[tiab] OR mpr[tiab] OR semiquantitative [tiab] OR semiquantitative [tiab] OR semiquantitative OR MPRI [tiab] OR myocardial blood flow [tiab] OR MBF [tiab] OR contrast enhancement ratio [tiab] OR left ventricular upslope [tiab] OR upslope integral [tiab] OR CER [tiab] OR SLP [tiab] OR INT [tiab]). Additionally, Embase and Web of Science were searched using adjusted search strategy to fit the search matrix of the source.

### Study selection

The search strategy was set-up in collaboration with the local Medical Library (Central Medical Library University Medical Center Groningen). One researcher (RvD) executed the search and gathered the results in Mendeley (version 1.16.1). A check for duplicates was performed with both the built in ‘*check for duplicates*’ function as well as manually (RvD). Screening for study eligibility and relevance of the articles retrieved by the search strategy was performed individually by two reviewers (RvD and MvA) using the pre-defined study eligibility criteria. Studies were categorized as includable, possibly includable, non-includable by screening the titles and abstracts. Inter-reviewer categorization was compared and in case of disagreement discussed to obtain consensus.

### Data collection process

The primary data extraction was performed independently by both researchers (RvD and MvA) with the use of a predefined template. Data extraction was cross checked and discussed to achieve consensus. In case of missing or unclear data the corresponding authors were contacted (*n* = 12), in absence of a response the studies were excluded.

### Data items

The following patient characteristics were collected: age, gender, prevalence of CAD, and coronary artery disease risk factors. Data on study design was collected, including: prospective/retrospective set up, number of patients enrolled, number of patients excluded, scanner type and manufacturer, stressor agent and dose, contrast agent, perfusion sequence, cardiac segmentation method, reference standard, outcome measures with reported sensitivity, specificity, negative predictive value, positive predictive. The number of true positives (TP), false positives (FP), true negatives (TN), and false negatives (FN) were derived directly from the article or calculated from the sensitivity and specificity reported in the articles. All the figures and tables in this article are original for this article.

### Quality assessment

Two reviewers (RvD and MvA) independently evaluated the study quality of the included studies using the ‘Quality Assessment of Diagnostic Accuracy Studies Tool’ (QUADAS-2) [23]. Risk of bias was assessed across all studies and within each individual study using RevMan software (version 5.3.5, Cochrane collaboration).

### Statistical analysis

The principal summary measures used to assess diagnostic accuracy were sensitivity, specificity, Diagnostic Odds Ratio (DOR), and AUC. In case studies performed multiple semi-quantitative or quantitative analyses we chose the maximal upslope parameter as a representative measure for semi-quantitative analysis and absolute MBF for quantitative analysis. Furthermore, transmural ratios were used when studies reported sub-endocardial, sub-epicardial, and transmural outcomes. When multiple tracer kinetic models were used for quantitative analysis, the Fermi model was selected. When both a semi-quantitative and a quantitative outcome, or both 1.5 T as well as 3.0 T were used, both outcomes were taken into account for the analysis.

The primary data synthesis was based on bivariate mixed-effects binary regression modeling. Sensitivity, specificity, and heterogeneity (using the Q-statistic and I^2^ index) were calculated and displayed in forest plots. Significant heterogeneity was defined as Q-statistic *p* < 0.10 and/or I^2^ > 50%. Separate subgroup forest plots were evaluated when >5 studies were available.

The Deeks’ funnel test was used to test for publication bias, with a value <0.05 indicative of publication bias or systematic difference between results of larger and smaller studies. The DORs were used to calculate the summary receiver operating curves (sROC). Based on the ROC curves the AUC was calculated. Data analysis was performed with STATA (version 13.0; STATA corporation, Lakeway Drive, College Station, Texas, USA).

## Results

**Fig. 1:**
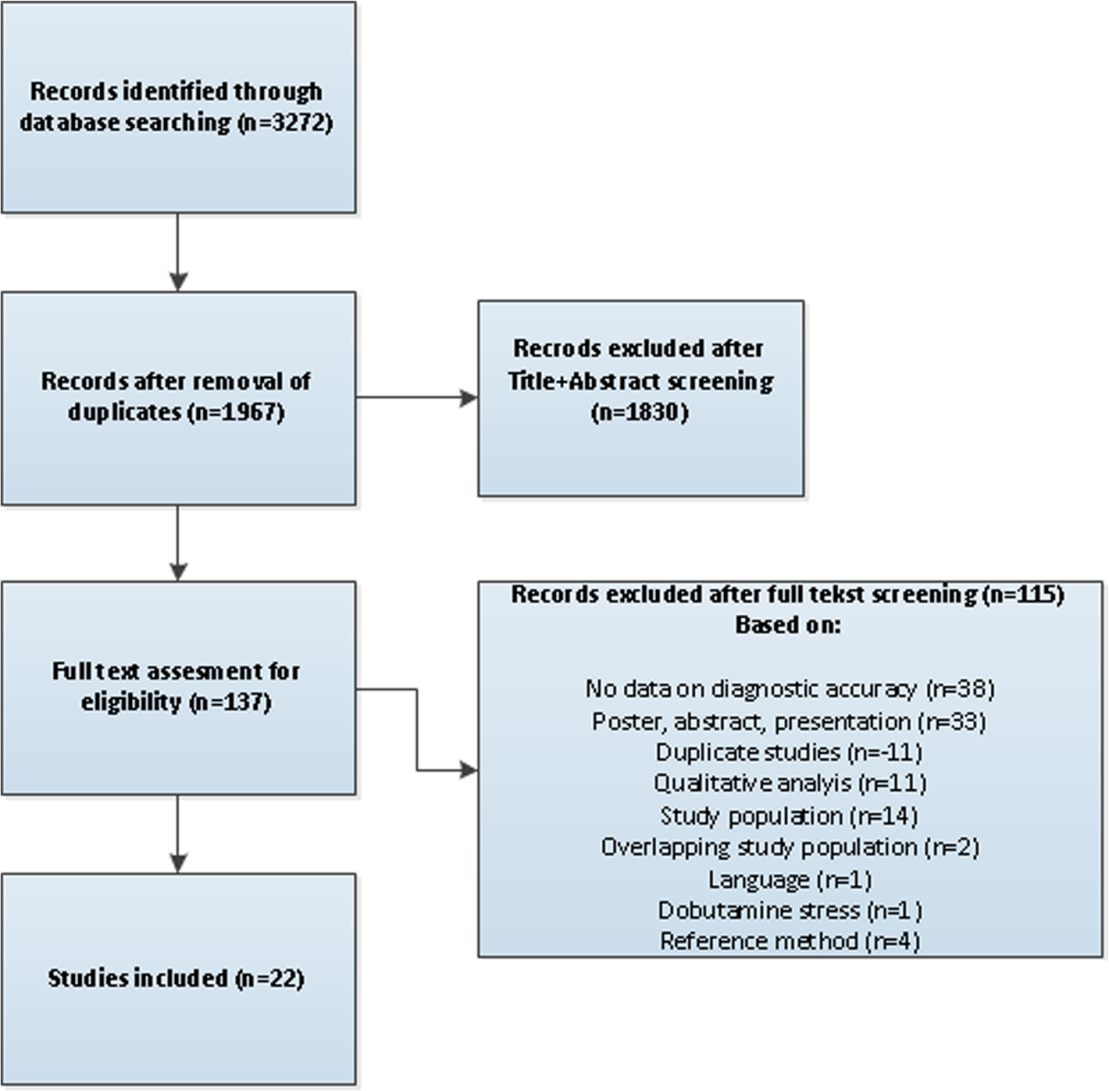
Flow diagram of the literature search and selection of relevant studies

The systematic search in Pubmed, WebOfScience, and Embase identified 3272 articles. After the removal of duplicates, 1967 articles were screened based on title and abstract. The resulting 137 articles were assessed in full text for eligibility. Of these, 23 articles were deemed eligible based on the predefined study eligibility criteria including a total of patients, with mean age ranging from 57 to 67 years. The PRISMA flowchart is shown in Fig. 1. The final analysis included 22 articles due to exclusion of one study using dobutamine as a stressor agent in which an inadequate heart rate response for diagnosis was achieved in most age groups.

Studies were performed at 1.5 T in 20 (91%) studies and at 3 T in 6 (27%) studies (Bernhardt et al. used both 1.5 T as well as 3.0 T). The stressor agent used was either adenosine or dipyridamole in 18 (82%) and 6 (27%), studies, respectively. Segment based outcome data was available in 4 (18%) of all studies, territory based outcome data was available in 13 (59%) and patient based outcome data in 11 (50%) studies included (Bertschinger et al. and Papanastasiou et al. reported both territory and patient based data). Perfusion analysis was performed semi-quantitative in 16 (73%) studies and quantitative in 10 (45%) (Huber et al. and Mordini et al. reported data on both semi-quantitative and quantitative analysis). The reference standard was anatomical in 15 (68%) studies and functional in 11 (50%). See Tables 1, 2 and 3.

**Table 1:** Overview of patient demographics for all included studies

Study	No. Patients	Male	Age^a^	HT (%)	DM (%)	smoking	Hypercholesterolemia (%)	History of PCI/CABG (5)	prevalence of CAD %	Previous MI (%)
Al-Saadi 2000 [27]	34	32	59+/−11	NS	NS	NS	NS	NS	100	NS
Bertschinger 2001 [28]	14	NS	NS	NS	NS	NS	NS	NS	93	NS
Ibrahim 2002 [29]	25	19	63+/−13	NS	28	NS	68	56	100	12
Nagel 2003 [30]	84	73	63+/−8	0	0	21	NS	NS	51	0
Giang 2004 [31]	29	25	58+/−8	45	14	34	59	52	66	38
Plein 2005 [32]	92	68	58+/−11	30	8	35	54	NS	64	19
Rieber 2006 [33]	43	38	66+/−8	86	23	35	NS	28	67	19
Positano 2006 [34]	32	20	65+/−10	NS	NS	NS	NS	NS	50	NS
Costa 2007 [35]	37	16	65+/−11	80	23	20	57	NS	97	NS
Pignitore 2008 [36]	125	51 14	62+/−7 60+/−5	73 78	27 26	51 59	70 66	NS	71	NS
KrittayaPhong 2009 [37]	66	38	61+/−12	62	27	8	62	Exclusion criterium	58	Exclusion criterium
Kirschbaum 2011 [38]	40	27	62+/−7	49	15	29	41	NS	34	NS
Lockie 2011 [39]	42	33	57+/−10	NS	19	21	Exclusion criterium	19	NS	Exclusion criterium
Bernhardt 2012 [25]	34	26	62+/−11	80	15	47	53	NS	62	NS
Huber 2012 [24]	23	27	67+/−12	36	23	85	29	NS	55	19
Motwani 2012 [40]	40	27	64+/−8	NS	NS	NS	NS	NS	53	NS
Chiribiri 2013 [41]	30	22	59+/−11	NS	27	27	NS	NS	80	NS
Mordini 2014 [20]	67	45	60+/−11	60	16	42	75	25	34	25
Motwani 2014 [42]	35	26	62+/−8	51	17	40	54	9	57	9
Yun 2015 [43]	58	17	60+/−11	59	26	28	48	10	31	16
Pan 2015 [44]	71	57	60+/−6	8	31	61	62	9	55	NS
Papanastasiou 2016 [45]	24	20	63 ± 7	13	3	6	NS	4	67	7

^a^Age either mean+/−SD or mean(range). *HT* hypertension, *DM* diabetes mellitus, *PCI* percutaneous coronary intervention, *CABG* coronary artery bypass graft, *CAD* Coronary Artery Disease, *MI* myocardial infarct

**Table 2:** Overview of the study specific acquisition protocol

Study	Scanner	Protocol	Stressor agent	Contrast agent	Contrast dosage	Perfusion sequence
Al-Saadi 2000 [27]	1.5 T, Philips	Rest/stress	Dipyridamole	Gadopentate (Magnevist)	0.025 mmol/kg	T1-weighted inversion recovery single-shot turbo gradient echo
Bertschinger 2001 [28]	1.5 T, G.E.	Stress only	Dipyridamole	Gadodiamide (Omniscan)	NS	interleaved gradient-echo EPI
Ibrahim 2002 [29]	1.5 T, Phillips	Rest/stress	Adenosine	Gadopentate (Magnevist)	0.05 mmol/l	A fast hybrid, gated-imaging sequence consisting of three short- axis slices was used
Nagel 2003 [30]	1.5 T, Philips	Rest/stress	Adenosine	Diethylenetriaminepentaacetic acid-gadolinium	0.025 mmol/kg	single shot segmented k-space turbo-gradient-echo/echo-planar-imaging (EPI)-hybrid
Giang 2004 [31]	1.5 T, G.E.	Stress only	Adenosine	Gadopentate (Magnevist)	0.05 mmol/kg	hybrid echo planar
Plein 2005 [32]	1.5 T, Philips	Rest/stress	Adenosine	Gadopentate (Magnevist)	0.05 mmol/kg	dynamic segmented k-space gradient-echo combined with SENSE
Rieber 2006 [33]	1.5 T, Siemens	Stress/rest	Adenosine	Gadodiamide (Omniscan)	0.05 mmol/kg	T1-weighted saturation recovery turbo flash
Positano 2006 [34]	1.5 G.E.	Rest/stress	Dipyridamole	Gadodiamide (Omniscan)	0.1 mmol/kg	fast gradient-echo train
Costa 2007 [35]	1.5 Siemens	Stress/rest	Adenosine	Gadolinium (Magnevist)	0.1 mmol/kg	single-shot gradient-echo
Pignitore 2008 [36]	1.5 G.E.	Rest/stress	Dipyridamole	Gadodiamide (Omniscan)	0.1 mmol/kg	fast gradient-echo train
KrittayaPhong 2009 [37]	1.5 T, Phillips	Stress/rest	adenosine	Gadopentate (Magnevist)	0.05 mmol/l	ECG-triggered, T1 weighted, inversion receovery single shot turbo gradient echo sequence
Kirschbaum 2011 [38]	1.5 T, GE Medical Systems	Rest/stress	adenosine	Gadopentate (Magnevist)	0.05 mmol/kg	steady state free-precession technique
Lockie 2011 [39]	3.0 T, Philips	Stress/rest	Adenosine	Gadopentate (Magnevist)	0.05 mmol/kg	saturation recovery gradient echo method
Bernhardt 2012 [25]	1.5 T/3.0 T, Philips	Stress/rest	Adenosine	Gadoterate meglumine (Dotarem)	0.075 mmol/kg	steady state free-precession technique
Huber 2012 [24]	1.5 T, Siemens	NS	Adenosine	Gadopentate (Magnevist)	0.05 mmol/kg	saturation turboFlash
Motwani 2012 [40]	3.0 Phillips	Stress/rest	Adenosine	Gadopentate (Magnevist)	0.05 mmol/kg	Saturation-recovery gradient echo
Chiribiri 2013 [41]	3.0 T, Philips	Stress/rest	Adenosine	Gadopentate (Magnevist)	0.05 mmol/kg	saturation-recovery gradient echo
Mordini 2014 [20]	1.5 T, Siemens	Stress/rest	Dipyridamole	Gadopentate (Magnevist)	0.005 mmol/kg followed by 0.1 mmol/kg	saturation recovery hybrid echo-planar
Motwani 2014 [42]	3.0 T, Philips	Stress/rest	Adenosine	Gadobutrol (Gadovist)	0.075 mmol/kg	3D spoiled turbo gradient-echo
Yun 2015 [43]	3.0 T, Philips	Stress/rest	Dipyridamole	Gadobenate Dimeglumine (Multihance)	0.05 mmol/kg	saturation recovery gradient-echo T1-weighted
Pan 2015 [44]	3.0 T, Siemens	Stress/rest	Adenosine	Gadobutrol (Gadovist)	0.075 mmol/kg	T1-weighted saturation recovery turbo flash
Papanastasiou 2016 [45]	3.0 T, Siemens	Stress/rest	Adenosine	Gadobutrol (Gadovist)	0.05 mmol/kg	Turbo-fast low saturation recovery single-shot gradient echo

**Table 3:** Overview of study specific cardiac segmentation method, data interpretation, reference standard, cut-off values for significant stenosis and semi-quantitative and/or quantitative analysis

Study	Segmentation	Data interpretation	Reference standard	Cut-off values	Outcome variables
Al-Saadi 2000 [27]	6 segments (mid ventricular)	Territory	QCA	≥75% DS	Semi-quantitative
Bertschinger 2001 [28]	4 × 8 segments	Patient/Territory	QCA	≥50% stenosis	Semi-quantitative
Ibrahim 2002 [29]	3 short axis slices 18 segments per slice/polar maps subdivided into 6 segments	Territory	QCA	>75% DS	Semi-quantitative
Nagel 2003 [30]	5 short axis slices 6 segments per slice	Patient	Visual ICA	≥75% DS	Quantitative
Giang 2004 [31]	3 × 8 segments good quality score	Patient	QCA	≥50% DS	Semi-quantitative
Plein 2005 [32]	16 segments (AHA)	Patient	Visual ICA	>70% DS	Quantitative
Rieber 2006 [33]	16 segments (AHA)	Territory	QCA + FFR	>50% DS on QCA and FFR ≤0.75	Semi-quantitative
Positano 2006 [34]	3 short axis slices 16 segments	Segment	QCA	≥75% DS	Semi-quantitative
Costa 2007 [35]	3 short axis 8 segments per slice	Segment	QCA	>70% DS	Quantitaive
Pignitore 2008 [36]	3 short axis slices 16 segments	Segment	QCA	≥50% DS	Semi-quantitative
KrittayaPhong 2009 [37]	16 segments (AHA)	Patient	Visual ICA	≥50%	Semi-quantitative
Kirschbaum 2011 [38]	16 segments (AHA)	Patient	ICA with CFR	CFR < 2.0	Semi-quantitative
Lockie 2011 [39]	16 segments (AHA)	Territory	FFR	<0.75	Quantitative
Bernhardt 2012 [25]	16 segments (AHA)	Patient	FFR	≤0.80	Semi-quantitative
Huber 2012 [24]	18 segments (6 per slice)	Territory	QCA + FFR	>75% DS on QCA or 51 - 75% DS on QCA + FFR <0.75	Semi-quantitative/Quantitative
Motwani 2012 [40]	1 midventricular slice 6 segments	Segment	QCA	>70% DS	Quantitative
Chiribiri 2013 [41]	16 segments (AHA)	Territory	FFR	<0.80	Quantitative
Mordini 2014 [20]	3 short axis slices 12 segments per slice	Patient	QCA	>70% DS	Semi-quantitative/Quantitative
Motwani 2014 [42]	Whole heart	Territory	QCA	≥75% DS	Quantitative
Yun 2015 [43]	16 segments (AHA)	Territory	QCA	>70% DS	Semi-quantitative
Pan 2015 [44]	16 segments (AHA) (mean of 2 lowest value assigned to coronary territories)	Territory	FFR	≤0.75	Quantitative
Papanastasiou 2016 [45]	16 segments (AHA)	Patient/Territory	ICA + FFR	≥70% DS on ICA or FFR <0.80 and luminal stenosis ≥50%	Quantitative

### Diagnostic performance

**Fig. 2:**
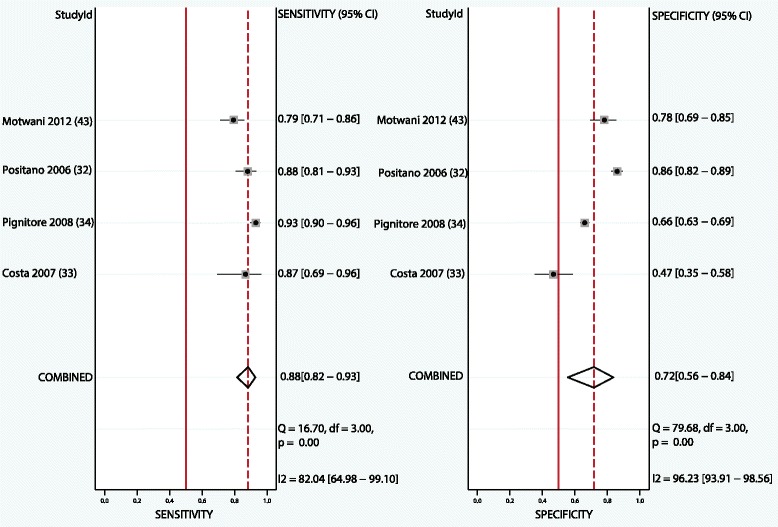
Forest plot of per segment sensitivity and specificity of both semi-quantitative and quantitative CMR perfusion analysis against anatomical and functional reference standards. Significant heterogeneity was defined as Q-statistic *p* < 0.10 and/or I^2^ > 50%

**Fig. 3:**
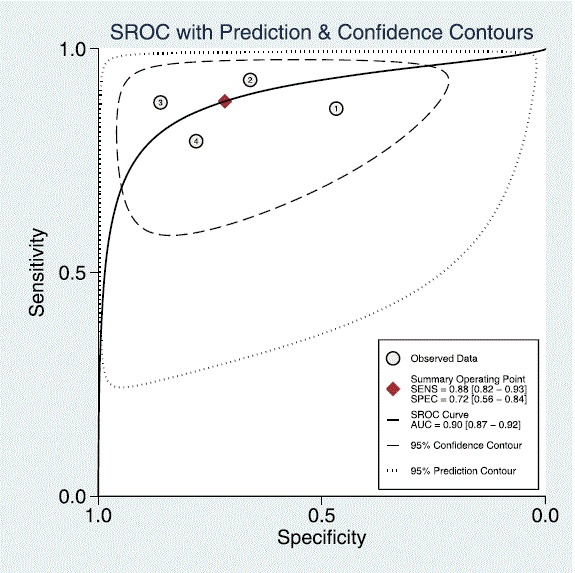
Summary receiver operating curve of the diagnostic performance of segmental semi-quantitative and quantitative CMR perfusion analysis

Four studies with per segment-based analysis could be included, all using an anatomical reference method (QCA). Segment-based pooled sensitivity, specificity, and DOR were 0.88 (95% CI, 0.82–0.93), 0.72 (95%CI, 0.56–0.84), and 19 (95% CI, 9–40), respectively. ROC curve analysis showed an AUC of 0.90 (95% CI, 0.87–0.92). See Table 4 and Figs. 2 and 3.

**Table 4:** Pooled diagnostic accuracy of semi-quantitative and quantitative CMR perfusion analysis on segmental, territory, and per patient basis (bold) and subgroup analysis of anatomical/functional reference standard or semi-quantitative/quantitative analysis (unbold)

	No. Studies	No. S/T/P	Sensitivity	Q-statistics *p*-value^a^	I^2b^	Specificity	Q-statistics *p*-value^a^	I^2b^	PLR	NLR	DOR	AUC
**Per Segment**	**4**	**3838**	**0.88 (0.82–0.93)**	**0.00**	**82.04**	**0.72 (0.56–0.84)**	**0.00**	**96.23**	**3.1 (1.0–5.10)**	**0.16 (0.10–0.26)**	**19 (9–40)**	**0.90 (0.870.92)**
**Per territory**	**12**	**1058**	**0.82 (0.77–0.86)**	**0.49**	**0.00**	**0.83 (0.74–0.90)**	**0.00**	**90.68**	**5.0 (3.1–7.9)**	**0.22 (0.17–0.29)**	**23 (12–44)**	**0.84 (0.81–0.87)**
Anatomical reference	5	370	0.85 (0.78–0.90)	0.49	0.00	0.83 (0.72–0.91)	0.00	78.11	5.1 (2.9–9.2)	0.18 (0.12–0.27)	28 (13–63)	0.86 (0.83–0.89)
Functional reference	7	688	0.77 (0.63–0.86)	0.00	86.70	0.85 (0.73–0.92)	0.00	93.19	5.1 (2.5–10.3)	0.28 (0.16–0.48)	18 (6–59)	0.88 (0.84–0.90)
Semi-quantitative	6	343	0.77 (0.60–0.88)	0.00	86.96	0.84 (0.76–0.89)	0.30	17.10	4.7 (2.9–7.8)	0.28 (0.15–0.53)	17 (6–50)	0.87 (0.84–0.90)
Quantitative	6	729	0.77 (0.62–0.87)	0.00	89.39	0.86(0.72–0.94)	0.00	94.92	5.5 (2.4–12.6)	0.27 (0.14–0.49)	21 (6–8)	0.88(0.85–0.91)
**Per patient**	**10**	**566**	**0.83 (0.75–0.88)**	**0.01**	**60.71**	**0.76 (0.65–0.85)**	**0.00**	**66.27**	**3.5 (2.2–5.5)**	**0.23 (0.14–0.36)**	**15 (6–36)**	**0.87 (0.84–0.90)**

^a^Q statistic *p*-value <0.10 and/or ^b^I^2^ > 50% is considered to indicate heterogeneity. Subgroup analysis was performed when ≥5 studies were available

**Fig. 4:**
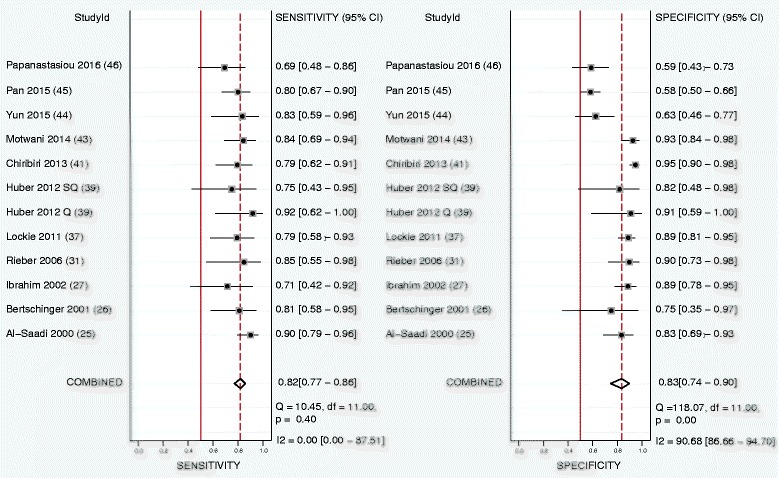
Forest plot of per territory sensitivity and specificity of both semi-quantitative and quantitative perfusion analysis against anatomical and functional reference standards. Significant heterogeneity was defined as Q-statistic *p* < 0.10 and/or I^2^ > 50%

**Fig. 5:**
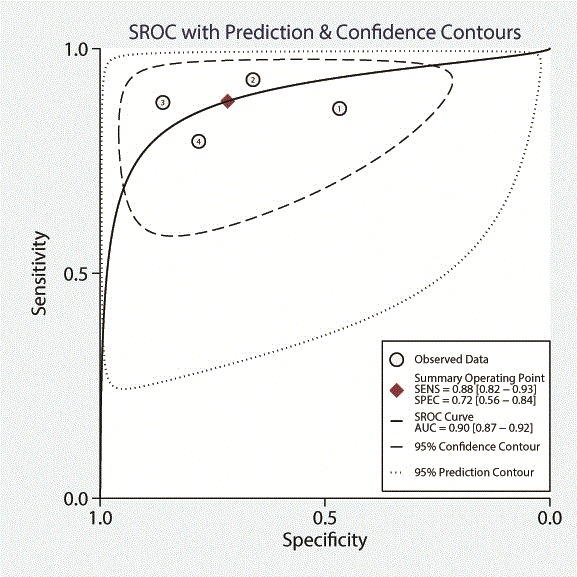
Summary receiver operating curve of the diagnostic performance of territory based semi-quantitative and quantitative CMR perfusion analysis

Eleven studies were included analyzing the perfusion data on a per territory basis with Huber et al. [24] reporting on both semi-quantitative and quantitative analysis, including twelve study outcomes in the final per territory analysis. Territory-based pooled sensitivity, specificity, and DOR were 0.82 (95% CI, 0.77–0.86), 0.83 (95% CI, 0.74–0.90), and 21 (95% CI, 10–45), respectively. ROC curve analysis showed an AUC of 0.84 (95% CI, 0.81–0.87). See Table 4 and Figs. 4 and 5 **.** Quantitative analysis (*n* = 6) on a per territory base yielded a sensitivity, specificity, and DOR of 0.77 (95% CI, 0.62–0.87), 0.86 (95% CI, 0.72–0.94), and 21 (95% CI, 6–8) with an AUC of 0.88 (95% CI, 0.85–0.91), while semi-quantitative analysis (*n* = 6) yielded a sensitivity and specificity of 0.77 (95% CI, 0.60–0.88) and 0.84 (95% CI, 0.76–0.89) with an AUC of 0.87 (95% CI, 0.84–0.90). Using a functional reference (*n* = 7) standard yielded a sensitivity, specificity, and DOR of 0.77 (95% CI, 0.63–0.86), 0.85 (95% CI, 0.73–0.92), and 18 (95% CI, 6–59) with an AUC of 0.88 (95% CI, 0.84–0.90), while the use of an anatomical reference (*n* = 5) showed sensitivity, specificity, and DOR of 0.85 (95% CI 0.78–0.90), 0.83 (95% CI, 0.72–0.91), and 28 (95% CI, 13–63) with an AUC of 0.86 (95% CI, 0.83–0.89).

**Fig. 6:**
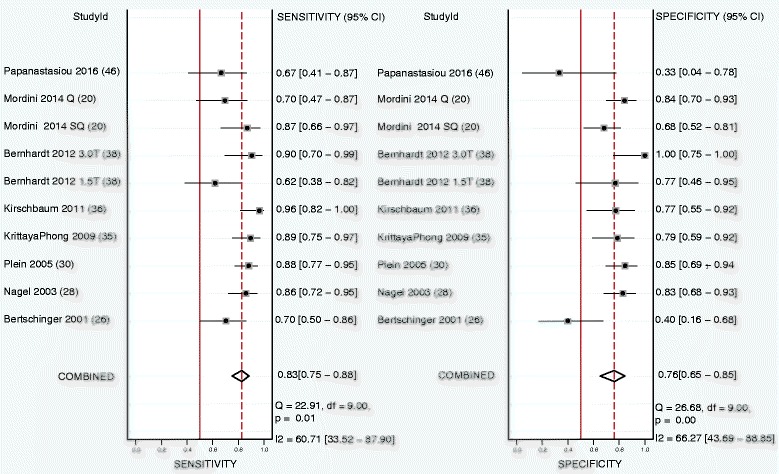
Forest plot of per patient sensitivity and specificity of both semi-quantitative and quantitative perfusion analysis against anatomical and functional reference standards. Significant heterogeneity was defined as Q-statistic *p* < 0.10 and/or I^2^ > 50%

**Fig. 7:**
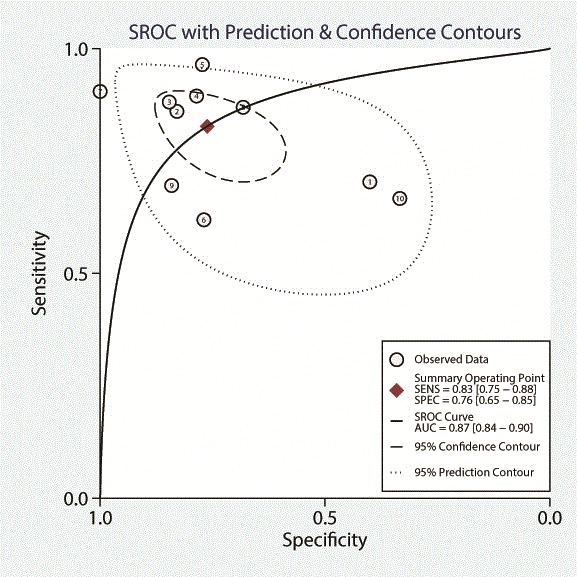
Summary receiver operating curve of the diagnostic performance of patient based semi-quantitative and quantitative CMR perfusion analysis

Eight studies were included analyzing the CMR perfusion data on a per patient basis, of which Mordini et al. [20] reported on both semi-quantitative and quantitative outcome and Bernhardt et al. [25] performed analysis at both 1.5 T and 3.0 T, in the end including ten study outcomes in the final per patient analysis. Six had an anatomical reference standard and 4 a functional reference standard. Patient based sensitivity, specificity, and DOR were 0.83 (95% CI, 0.75–0.88), 0.76 (95% CI, 0.65–0.85), and 15 (95%CI 6–36). ROC curve analysis showed an AUC of 0.87 (95% CI, 0.84–0.90). See Table 4 and Figs. 6 and 7.

### Diagnostic accuracy in patients with decreased left ventricular ejection fraction or multi-vessel disease

The study of Krittayaphong et al. reported on the diagnostic accuracy of MPRI in patients with decreased left ventrticular ejection fraction (LVEF). They report a decreased sensitivity, specificity and diagnostic accuracy in the subgroup of patients with decreased LVEF (sensitivity 88.9%, specificity 58.3% and diagnostic accuracy 71.5) as compared to patients with normal LVEF (sensitivity 89.7%, specificity 93.8% and diagnostic accuracy 91.1). Mordini et al. report that all their patients with multivessel disease (*n* = 7) were correctly identified with quantitative perfusion analysis. Giang et al. present a similar sensitivity and specificity whether patients with three vessel disease were included or not across all tested doses (e.g. 94/71% sensitivity/specificity when patients with three vessel disease included at dose 3 compared to a 91%/71% sensitivity/specificity when patients with three vessel disease excluded).

### Study quality assessment and publication bias

**Fig. 8:**
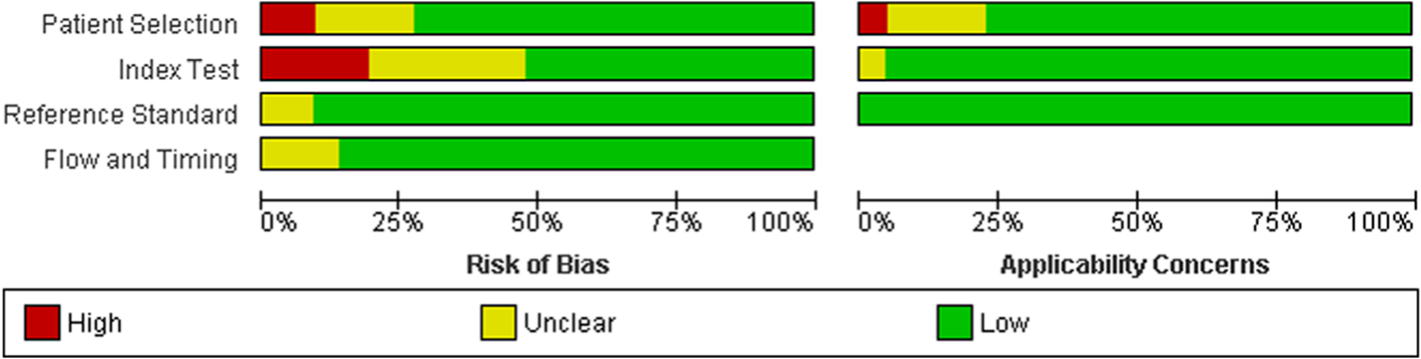
Deeks’ funnel plots of the studies on per segment (**a**), per territory (**b**), and per patient (**c**) basis. *P*-value <0.05 indicative of publication bias or systematic difference between results of larger and smaller studies

**Fig. 9:**
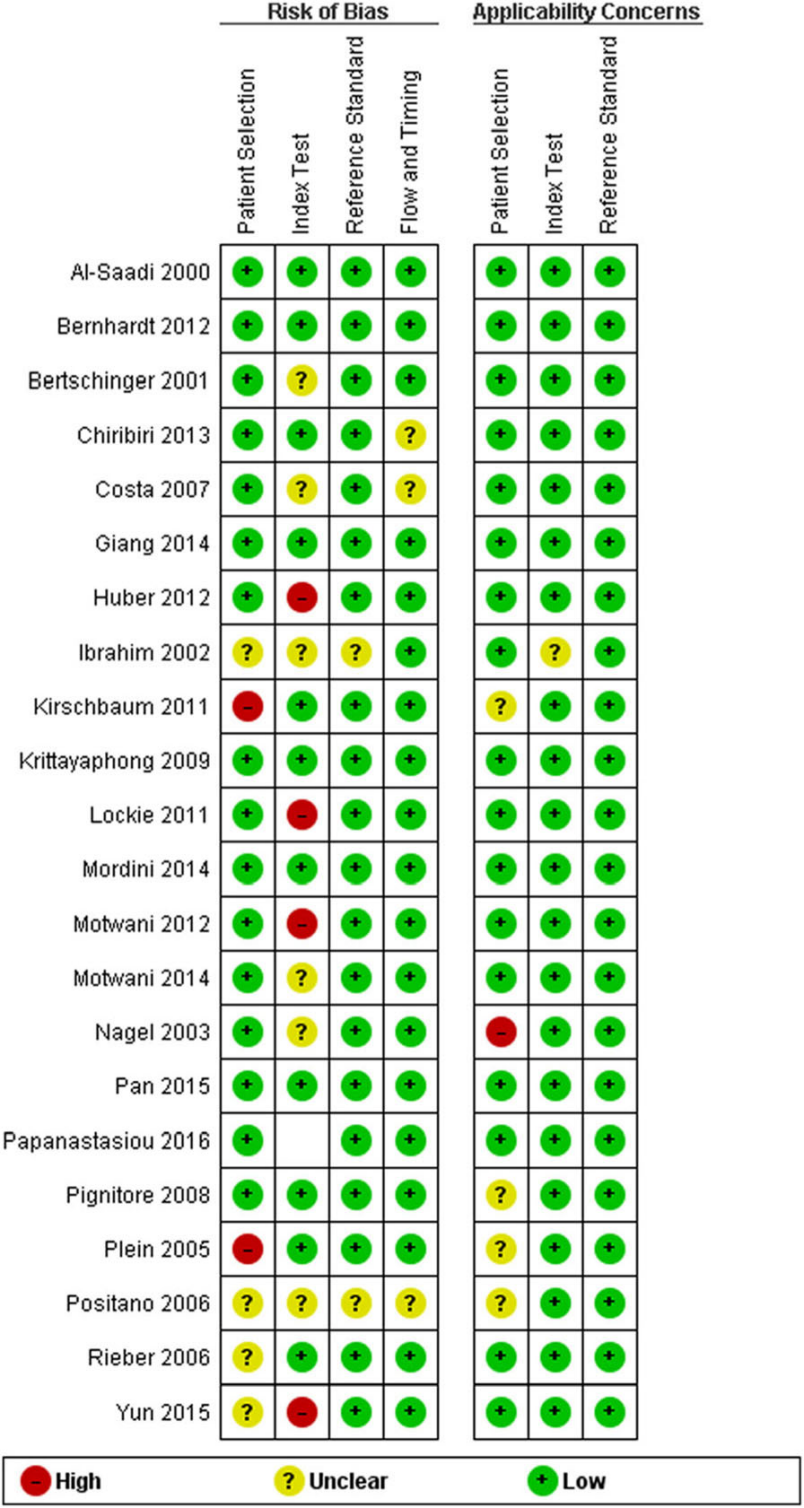
Deeks’ funnel plots of the subgroup analysis on per territory basis with anatomical reference standard (**a**), functional reference standard (**b**), semi-quantitative analysis (**c**), and quantitative analysis (**d**). *P*-value <0.05 indicative of publication bias or systematic difference between results of larger and smaller studies

**Fig. 10:**
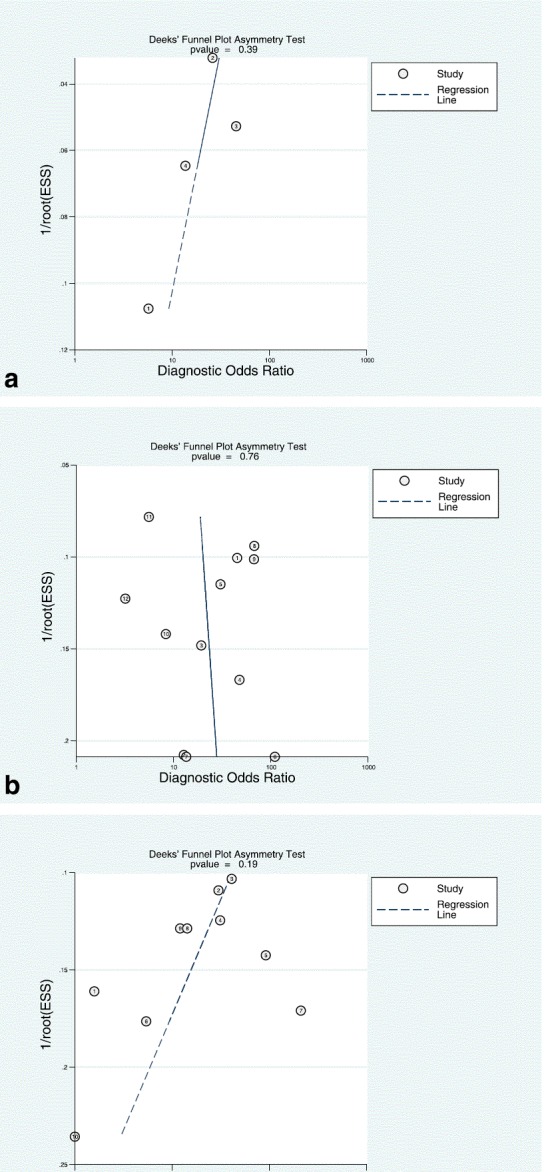
Summary of the risk of bias and applicability concerns across the included studies as assessed with QUADAS-2 forms by the reviewers

**Fig. 11:**
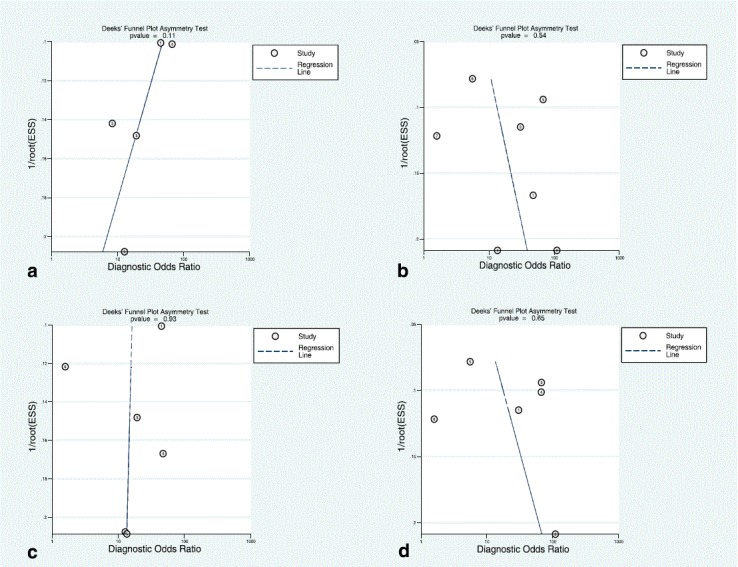
Risk of bias and applicability concerns assessment with an overview of the reviewers judgment about each separate domain for each included study

The overall methodological quality of the studies was good See Figs. 8 and 9. The per territory analysis pooled sensitivity, per territory anatomical reference standard sensitivity and per territory semi-quantitative specificity did not show significant heterogeneity See Figs. 2, 4, 6 and Table 4. The Deeks’ Funnel plots did not indicate publication bias or systematic difference between results of larger and smaller studies See Figs. 10 and 11.

## Discussion

### Summary of evidence

The pooled diagnostic accuracy for segment-, territory- and patient-based analyses showed good diagnostic performance. The diagnostic accuracy of CMR perfusion analysis has been assessed in previous meta-analyses [13, 15–18]. However, this meta-analysis is the first focusing on the semi-quantitative and quantitative analysis of the SI-curves. The diagnostic accuracy of CMR perfusion (pooled for visual, semi-quantitative, and quantitative analysis) reported in the earlier meta-analyses range from AUC 0.90 to 0.94 [13, 15–18]. When comparing our results, the SI-curve based analysis of CMR perfusion does not lead to an increase in the diagnostic accuracy as compared to the combined diagnostic accuracy of CMR perfusion as reported in these previous papers. Visual analysis of CMR perfusion does not yield lower diagnostic accuracy. This is possibly due to the fact that visual observations are made upon fewer and less complex assumptions than both the semi-quantitative and quantitative analysis methods that are used. Both semi-quantitative and quantitative perfusion analysis are based on SI-curves and calculate a derivative of myocardial blood flow based on certain assumptions. The models used for quantitative analysis are mathematical representations of a physiological process and rely on assumptions made about the dynamic of contrast and blood plasma and pre-existing knowledge about the physiologic process and model dynamics. In these models it is assumed that there is no diffusion of contrast medium into the intracellular space. Unfortunately, only in a few specific contrast agents this is the case. Different models are used for CMR perfusion analysis, with different degrees of complexity, and the optimal model is yet to be determined. The complexity of this modeling process, the many assumptions made and thereby the selection of a suitable model makes model-dependent perfusion analysis highly susceptible to error and with inconsistent results as a consequence. The use of different models with varying results could add to the heterogeneity in the quantitative analysis group. Semi-quantitative analysis, although in theory inferior to quantitative analysis, is a relatively simple method to estimate perfusion. The low complexity of these methods make it a robust method, allowing for less variation among research groups. As visual CMR perfusion analysis is relatively simple as compared to either semi-quantitative and/or quantitative CMR perfusion analysis, it is possible that this method is less susceptible to methodological errors (causing false conclusions). However, the methods used for assessing semi-quantitative CMR perfusion also vary within studies. The large variation in both semi-quantitative and quantitative CMR perfusion post processing techniques make it challenging to make an accurate comparison due to extensive inter-study heterogeneity. We compared the diagnostic accuracy of semi-quantitative and quantitative CMR perfusion analysis on a per territory basis and observed that the diagnostic accuracy slightly decreased using quantitative analysis (AUC of 0.87(0.83–0.89) compared to 0.81(0.78–0.85)). This is possibly due to the fact that quantitative analysis is based on multiple assumptions.

If the noninvasive MPI techniques are to be used as a gatekeeper for further diagnosis and treatment it is important to select a modality in which the amount of false negative results is low to assure that patients with significant disease are not missed. This requires the sensitivity of the gatekeeper test to be high. We were also performed subgroup analyses in the per territory group, based on the reference standards used. The anatomical reference standards merely depict the presence or absence of epicardial coronary stenosis (visual invasive coronary angiography, QCA), whereas the functional reference standards contained functional information on either pressure drop across the stenosis (FFR).

Our results show similar diagnostic accuracy when anatomical reference standards were used (0.85(0.82–0.88)) as compared to the diagnostic accuracy of SI-curve analysis with the use of functional reference standards (0.82(0.79–0.86)) in the per territory analysis.

For the anatomical reference standard, a DS >50, >70% or >75% were generally used as the cut-off value for significant CAD in both QCA and visual angiographic assessment. For the functional reference standard, a FFR of either <0.75 or <0.8 were used to indicate significant CAD. The accuracy of the anatomical reference standards as well as the currently used gold standard for functional reference of invasive coronary angiography +/− FFR for determining flow limiting CAD are debatable. Furthermore, pooling of the different threshold also increases heterogeneity in this meta-analysis. Previous research has shown that the anatomical presence of a stenosis, with cut-off values of either >50% DS or >70% DS have a poor correlation with FFR [1]. The use of the functional FFR measurement to guide therapy has proven to be superior as compared to anatomical assessment alone [2]. The FFR measurement is based on the measurement of a pressure drop across an epicardial vessel pre- and post-stenosis and a value of either <0.75 or a more liberal cut-off of <0.8 is used to indicate a functionally significant epicardial stenosis. However, what both the anatomical reference standard and the functional FFR measurement ignore microvasculature perfusion defects and the assumptions of a linear relationship between increasing stenosis or decreasing pressure with decreasing flow is made. To better understand the myocardial perfusion, van de Hoef et al. aimed to determine the relationship between invasively measured FFR and coronary flow reserve. The results of this study indicate a non-linear relationship between FFR (pressure drop information) and coronary flow reserve (flow information). The authors conclude that the disagreement between FFR and coronary flow reserve is caused by the involvement of the microvasculature and this indicates that the functional FFR measurement is not an accurate representation of myocardial perfusion [26]. We believe that there is a trend towards a better understanding of the complex process of myocardial perfusion and that the currently used reference standard as of yet fail to accurately represent myocardial perfusion. The need for a well validated and robust measurement technique for measuring myocardial perfusion is necessary and this technique might be used in the future as the gold standard. The inability of both the anatomical and functional reference standards to accurately represent myocardial blood flow might have influenced the results and so the results of this meta-analysis should be interpreted with caution.

Further research is necessary to determine the ideal golden standard for myocardial perfusion. We emphasize that it might be beneficial to first critically review phantom or ex-vivo research regarding the determination of myocardial perfusion in search for the measurement which represents true myocardial blood flow as accurately as possible.

In our meta-analysis we found an extensive variation in study population, CMR protocols, post processing techniques, and reference standards used. The lack of standardized CMR perfusion protocols or post processing techniques might have influenced our estimates of a lower diagnostic accuracy than expected of semi-quantitative and quantitative CMR perfusion analysis as compared to visual assessment. The extensive heterogeneity between the study protocols should be taken into account in the interpretation of these results. Standardization of the analysis protocols is needed to make more generalizable recommendations.

Future research should focus on the construction of a quantitative model that accurately depicts physiological myocardial blood flow. The different quantitative models should be compared and validated within a well-structured standardized CMR perfusion protocol preferably against a well validated perfusion method to determine which of the models accurately describes the perfusion process. Specific cut-off values to distinguish between normal and ischemic myocardium should be determined, and CMR protocols should be calibrated between the different CMR scanners. Visual CMR perfusion analysis alone is already highly accurate in the assessment of significant CAD and might also benefit from standardization of CMR protocols. The included studies reported results per segment, vessel territory or per patient. In this study we chose to include all three groups and report the results separately. However, it should be noted that a per segment based analysis holds more anatomical value since CAD often involves only specific coronary branches and not an entire vessel, affecting an entire vessel territory. This could have resulted in a lower diagnostic accuracy for the territory based results. The per territory analysis however, has a high clinical value since intervention more likely target the main coronary vessels instead of the secondary branches.

### Limitations

The main limitations for this meta-analysis is the small number of studies available regarding either segment, territory or patient based semi-quantitative or quantitative analysis of SI-curves in the assessment of myocardial perfusion using CMR and the wide variety of CMR protocols used in these studies. This resulted in a high degree of heterogeneity and possible bias making inter-study comparison difficult. Furthermore, there was an overrepresentation of male patients in the included studies. This limitation makes the findings less generalizable for women. We also decided not to include visual CMR perfusion analysis as the diagnostic accuracy of this assessment has been assessed in previous meta-analyses and our aim was to explore the diagnostic accuracy of SI-curve based assessment.

Another limitation regarding this meta-analysis are the wide variety of reference standards used. We decided to pool all reference standards used to provide a more complete overview of the evidence regarding SI-curve analysis during CMR perfusion. For our subgroup analysis we decided to group reference standards on either providing anatomical or functional information and observed a difference in diagnostic accuracy when using either anatomical or functional reference standards.

We conclude that the reference standard used has an influence on the diagnostic accuracy of SI-curve CMR-perfusion analysis and discussed the unclear relationship of both currently used anatomical and functional reference standards with myocardial flow and perfusion.

### Conclusions

This meta-analysis provides an overview of 23 original studies reporting on the diagnostic accuracy of semi-quantitative or quantitative analysis of stress CMR perfusion on a per segment, per territory or per patient basis for the assessment of significant CAD. Based on our results we conclude that due to a high degree of inter-study heterogeneity the real value of signal intensity curve based analyses of stress CMR perfusion still remains unclear. Semi-quantitative analysis showed a higher diagnostic accuracy for per territory analysis in this meta-analysis, possibly because it is less complex and less susceptible to false assumptions during the calculation. However, quantitative analysis still shows the potential to be used for absolute quantification of myocardial blood flow and further studies should be performed to determine the quantitative model that best represent true myocardial blood flow. The standardization and validation of semi-quantitative or quantitative stress CMR perfusion is necessary before it can be safely implemented in clinical practice.

## Abbreviations

AUC: Area under the curve
CAD: Coronary artery disease
CMR: Cardiovascular magnetic resonance
CT: Computed tomography
DOR: Diagnostic odds ratio
FFR: Fractional flow reserve
FN: False negative
FP: False positive
LVEF: Left ventricular ejection fraction
MBF: Myocardial blood flow
MPI: Myocardial perfusion imaging
PET: Positron emission tomography
QCA: Quantitative coronary angiography
SI: Signal intensity
SPECT: Single-photon emission computed tomography
sROC: Summary receiver operating curve
TAC: Tissue attenuation curve
TN: True negative
TP: True positive
