# Correction to: Diagnostic performance of semi-quantitative and quantitative stress CMR perfusion analysis: a meta-analysis

**DOI:** 10.1186/s12968-017-0421-z

**Published:** 2018-01-04

**Authors:** Journal of Cardiovascular Magnetic Resonance

**Affiliations:** London, UK

## Correction

In the original publication of this article there was an error in Figs. 8, 9, 10 and 11. During typesetting the Figs. 8, 9, 10 and 11 have been incorrectly swapped. In this “publisher correction” the correct and the incorrect figures are published. The original publication has been updated. BioMed central apologizes to the authors and readers for any inconvenience caused.

**Fig. 8:**
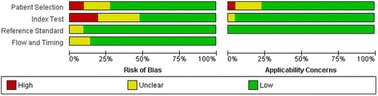
The original publication of Figure 8 with the caption “*Deeks’ funnel plots of the studies on per segment (****a****), per territory (****b****), and per patient (****c****) basis.* *P-value <0.05 indicative of publication bias or systematic difference between results of larger and smaller studies*”

**Fig. 8:**
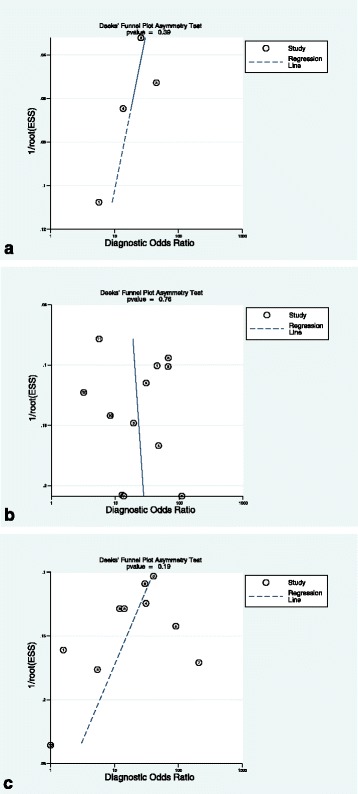
The corrected publication of Figure 8. with the caption *“Deeks’ funnel plots of the studies on per segment (****a****), per territory (****b****), and per patient (****c****) basis. P-value < 0.05 indicative of publication bias or systematic difference between results of larger and smaller studies”*

**Fig. 9:**
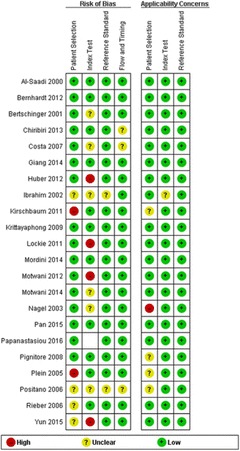
The original publication of Figure 9 with the caption: “*Deeks’ funnel plots of the subgroup analysis on per territory basis with anatomical reference standard (****a****), functional reference standard (****b****), semi-quantitative analysis (****c***), and quantitative analysis (***d***). *P*-value < 0.05 indicative of publication bias or systematic difference between results of larger and smaller studies.”

**Fig. 9:**
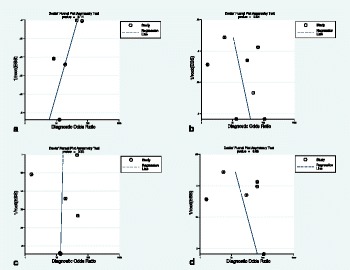
The corrected publication of Figure 9 with the caption: *“Deeks’ funnel plots of the subgroup analysis on per territory basis with anatomical reference standard (****a****), functional reference standard (****b****), semi-quantitative analysis (****c****), and quantitative analysis (****d****).* *P-value < 0.05 indicative of publication bias or systematic difference between results of larger and smaller studies”*

**Fig. 10:**
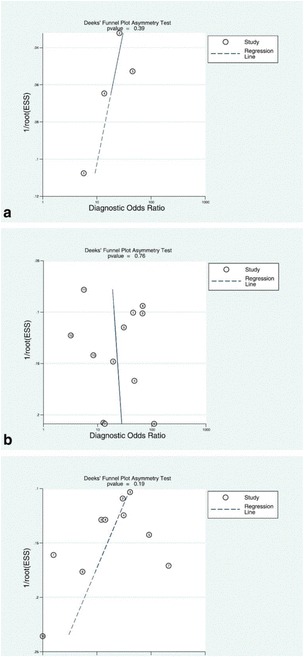
The original publication of Figure 10 with the caption: “ and applicability concerns across the included studies as assessed with QUADAS-2 forms by the reviewers”.

**Fig. 10:**
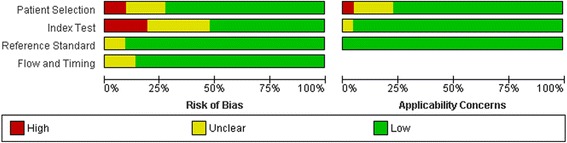
The corrected publication of Figure 10 with the caption*: “Summary of the risk of bias and applicability concerns across the included studies as assessed with QUADAS-2 forms by the reviewers”*

**Fig. 11:**
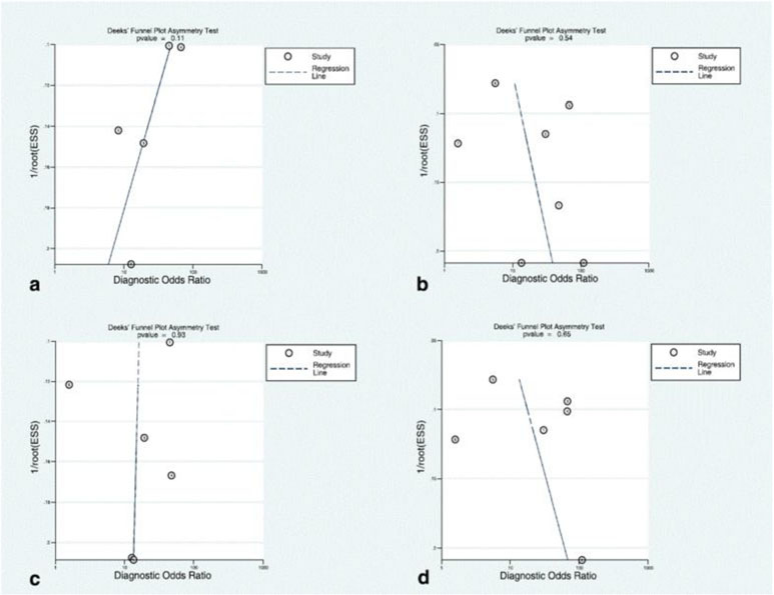
The original publication of Figure 11 with the caption: “Risk of bias and applicability concerns assessment with an overview of the reviewers judgment about each separate domain for each included study”

**Fig. 11:**
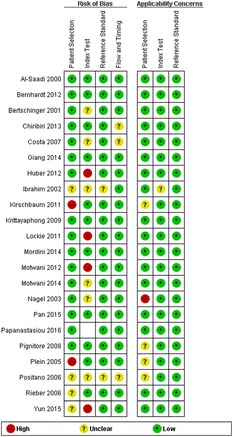
The corrected publication of Figure 11 with the caption*: “Risk of bias and applicability concerns assessment with an overview of the reviewers judgment abeout each separate domain for each included study”*

## References

[1] van Dijk, R., van Assen, M., Vliegenthart, R. et al. J Cardiovasc Magn Reson (2017) 19: 92. 10.1186/s12968-017-0393-z 10.1186/s12968-017-0393-z PMC5702972 29178905

